# Using *Caenorhabditis elegans* to fight human neurodegenerative diseases

**DOI:** 10.1186/1750-1326-8-S1-P12

**Published:** 2013-09-13

**Authors:** Xi Chen, Brian Kraemer, Jeff Barclay, Robert Burgoyne, Alan Morgan

**Affiliations:** 1 Department of Cellular and Molecular Physiology, Physiological Laboratory, Institute of Translational Medicine, University of Liverpool, Crown St, Liverpool, L69 3BX, UK; 2 Geriatric Research Education and Clinical Center, Veterans Affairs Puget Sound Health Care Systemepartment of Medicine, University of Washington, Seattle, WA, USA

## Background

Debilitating neurodegenerative disorders (NDs) including Alzheimer’s disease, Parkinson’s disease, and polyglutamine diseases are a major public health challenge in increasingly aging societies. Currently approved therapeutics are successful in slowing the progression of NDs but not in reversing or preventing the symptoms of NDs. The simplicity and amenability of the nematode *Caenorhabditis elegans* (*C*. *elegans*) to high-throughput genomic, proteomic and drug screening approaches make this organism an attractive choice for identifying new therapeutic compounds for these disorders and for understanding their mechanism of action. Indeed, a diverse set of informative *C. elegans* ND models have been developed manifesting abnormal behavioural or pathological phenotypes that partially recapitulate the salient cellular, molecular and pathological aspects of several distinct human NDs processes. Recent studies have identified *dnajc5* encoding cysteine-string protein α (CSPα) as the disease-causing gene of a rare autosomal dominant human ND known as adult-onset neuronal ceroid lipofuscinosis (ANCL). The null animal models of CSP are characterised by impaired neurotransmission, pre-synaptic neurodegeneration and premature mortality. Simple model organisms may therefore shed light on potential therapeutic approaches for ANCL and other NDs.

## Materials and methods

In this study, we are integrating multiple well-defined *C. elegans* ND models to uncover potential therapeutic interventions that target shared pathogenic pathways. We tested parameters such as locomotion and lifespan which act as a measure of neurological and neuromuscular function, and viability upon drug treatment in *C. elegans.*

## Results

Using locomotion behaviour and lifespan as phenotypic readouts, we uncovered the anticonvulsant ethosuximide as a promising candidate drug that not only rescues the short lifespan of a *C. elegans* null mutant model of ANCL, but also ameliorates the mobility defect and short lifespan of a distinct worm tauopathy model based on transgenic expression of mutant human tau. We are currently investigating how ethosuximide might exert its neuroprotective properties. We found that ethosuximide has no obvious aggregation-suppressing effect and suppression of proteotoxicity by ethosuximide is independent of the low voltage activated T-type calcium channel, the principal therapeutic target of ethosuximide in controlling generalised absence epilepsy.

## Conclusions

Overall, our data provide good evidence that anti-epileptic ethosuximide is efficacious in several worm ND models. Further investigation of its precise mode of action and validation in vertebrate systems is required to gauge the effectiveness of this compound as early leads for drug discovery. These findings should encourage further screening and characterisation of other neuroprotective compounds, and ultimately may assist in accelerating the clinical evaluation and development of drugs to combat protein conformational disorders in general.

